# Supplementary material for: Resolving the full spectrum of human genome variation using Linked-Reads
Source: Genome Res. 2019 Apr;29(4):635–45. doi: 10.1101/gr.234443.118 (PMC6442396; doi:10.1101/gr.234443.118)
Supplement: Supplemental Material [file supp_gr.234443.118_Supplemental_longranger-2.2.zip › longranger-2.2.tar.bz2!/lr2.2-public/lib/stan/cmdstan/stan/doc/api/html/functions_func_b.html]

Stan: Class Members - Functions


|  |  |
| --- | --- |
|  | Stan  2.5.0  probability, sampling & optimization |


- Main Page
- Related Pages
- Namespaces
- Classes
- Files

- Class List
- Class Index
- Class Hierarchy
- Class Members

- All
- Functions
- Variables
- Typedefs
- Enumerator
- Related Functions

- a
- b
- c
- d
- e
- f
- g
- h
- i
- j
- k
- l
- m
- n
- o
- p
- q
- r
- s
- t
- u
- v
- w
- x
- z
- ~


All Classes Namespaces Files Functions Variables Typedefs Enumerations Enumerator Friends Macros Pages

### - b -

- bare()
  : stan::io::cmd\_line
- bare\_size()
  : stan::io::cmd\_line
- bare\_type\_grammar()
  : stan::gm::bare\_type\_grammar< Iterator >
- base\_adapter()
  : stan::mcmc::base\_adapter
- base\_hamiltonian()
  : stan::mcmc::base\_hamiltonian< M, P, BaseRNG >
- base\_hmc()
  : stan::mcmc::base\_hmc< M, P, H, I, BaseRNG >
- base\_integrator()
  : stan::mcmc::base\_integrator< H, P >
- base\_leapfrog()
  : stan::mcmc::base\_leapfrog< H, P >
- base\_mcmc()
  : stan::mcmc::base\_mcmc
- base\_nuts()
  : stan::mcmc::base\_nuts< M, P, H, I, BaseRNG >
- base\_static\_hmc()
  : stan::mcmc::base\_static\_hmc< M, P, H, I, BaseRNG >
- base\_var\_decl()
  : stan::gm::base\_var\_decl
- base\_variable\_declaration()
  : stan::gm::arg\_decl
- basic\_validate()
  : stan::gm::validate\_var\_decl\_visgen
- begin\_update\_p()
  : stan::mcmc::base\_leapfrog< H, P >
  , stan::mcmc::expl\_leapfrog< H, P >
- BFGSLineSearch()
  : stan::optimization::BFGSLineSearch< M, QNUpdateType, Scalar, DimAtCompile >
- BFGSMinimizer()
  : stan::optimization::BFGSMinimizer< FunctorType, QNUpdateType, Scalar, DimAtCompile >
- binary\_op()
  : stan::gm::binary\_op
- boolean()
  : stan::json::json\_data\_handler
  , stan::json::json\_handler
- build\_tree()
  : stan::mcmc::base\_nuts< M, P, H, I, BaseRNG >
- bytes\_allocated()
  : stan::memory::stack\_alloc


---

[ Stan Home Page ]

*© 2011–2014,
Stan Development Team.*
